# Association between relative grip strength and depression among U.S. middle-aged and older adults: results from the NHANES database

**DOI:** 10.3389/fpubh.2024.1416804

**Published:** 2024-07-29

**Authors:** Aochuan Sun, Zhengtang Liu

**Affiliations:** ^1^The Department of Geriatrics, Xiyuan Hospital, China Academy of Chinese Medical Sciences, Beijing, China; ^2^Graduate School, Beijing University of Chinese Medicine, Beijing, China

**Keywords:** relative grip strength, depression, L-shaped, cross-sectional study, muscle strength, NHANES

## Abstract

**Background:**

Mental health issues among middle-aged and older adults are gaining increasing attention. Recent studies have shown that relative grip strength is associated with cardiovascular diseases and various cancers, but its relationship with depression remains unclear.

**Methods:**

This cross-sectional study included data from adults aged 50 years and older from the 2011–2014 National Health and Nutrition Examination Survey. Relative grip strength is calculated by dividing the maximum absolute grip strength of both hands by BMI. The Patient Health Questionnaire (PHQ-9) was used to evaluate the depressive outcome. Multivariate logistic regression was performed to assess the association between relative grip strength and depression.

**Results:**

In this study, a total of 3,639 participants (≥50 years) with a mean age of 64.3 ± 9.3 years were enrolled, of whom 48.9% were male. Compared with individuals with lower relative handgrip strength in Q1 (≤1.64 kg/BMI), the adjusted OR values for relative handgrip strength and depression in Q2 (1.64–2.17 kg/BMI), Q3 (2.17–2.84 kg/BMI), and Q4 (≥2.84 kg/BMI) were 0.69 (95% CI: 0.51, 0.93, *p* = 0.016), 0.36 (95% CI: 0.24, 0.55, *p* < 0.001), and 0.32 (95% CI: 0.20, 0.51, *p* < 0.001), respectively. The relationship between relative grip strength and depression presented an L-shaped curve (nonlinear, *p* = 0.006), with an inflection point of roughly 2.98 kg/BMI. Among participants with relative grip strength < 2.98 kg/BMI, the OR of incident depression was 0.41 (95% CI: 0.30–0.55, *p* < 0.001).

**Conclusion:**

Our findings indicated that relative grip strength was inversely associated with incident depression and demonstrated an L-shaped relationship among U.S. middle-aged and older adults. Relative grip strength could be the indicator for future screening of mental health.

## 1 Introduction

Depression is a common chronic medical condition that can affect both mental and physical health (1). The global prevalence of depression among the older adults is 31.74%, and this rate increases with age (2, 3). In older adults individuals, depression reduces the effectiveness of treatments for various chronic diseases and increases mortality rates (4). Therefore, based on the serious impact of geriatric depression and its prevalence in middle-aged and older adults people, it is very important to find indicators that can predict geriatric depression and intervene in advance.

Recent population-based studies have shown that low grip strength is associated with an increase in new-onset cognitive dysfunction (5), a 10-year risk of cardiovascular disease (6), and health-related quality of life decline (7). Previous research has found that lower grip strength reflects poorer physical activity overall, and is associated with tiredness, fear of falling, a sense of helplessness, and decreased social activity, all of which are risk factors for depressive symptoms (8, 9). Previous studies from multiple countries and ethnicities have found a negative correlation between grip strength and the incidence of depression (10–14). Furthermore, higher levels of grip strength can reduce the likelihood of depression among older adults populations with multiple comorbidities (15). However, since grip strength is strongly positively correlated with body size (16), absolute grip strength may not be suitable for older adults individuals with smaller body sizes (17–19). Thus, relative grip strength, which is absolute grip strength normalized according to body mass index, has been suggested to adjust the confounding of body size (20, 21). Most studies seem to consistently conclude that low relative grip strength is a risk factor for various chronic diseases (21–23). However, the effect of relative grip strength on the development of depression is still in dispute.

The objective of our study was to examine the association of relative grip strength with the incidence of depression in middle-aged and older Americans to fill these knowledge gaps. The secondary goal was to assess the dose–response relationship between relative grip strength and incident depression.

## 2 Materials and methods

### 2.1 Study participants and data sources

The National Health and Nutrition Examination Survey (NHANES) is a series of health-related studies intended to assess the health and nutritional condition of the non-institutionalized United States population using a multistage probability survey with stratification. The survey conducts an in-person home interview to capture demographics and information on medical history. Examination data, consisting of physiological, laboratory, and anthropometric data, were collected in the Mobile Examination Center (MEC). This project was approved by the National Center for Health Statistics Research Ethics Review Board, and all participants have written informed consent forms completed in advance of participation. According to the relevant policies, the secondary analysis was not subject to review by the Institutional Review Board.

Since grip strength measurement data were only available in the 2011–2014 NHANES database, we analyzed data from participants during this period. Our study’s participants were over 50 years old and had completed an interview and evaluation. Participants were excluded from the study if they had missing handgrip strength, Patient Health Questionnaire scores, or covariates.

### 2.2 Outcome ascertainment

The Patient Health Questionnaire (PHQ-9) was included as part of the MEC interview questionnaire to assess the depressive status of participants in the NHANES database who met the inclusion criteria. Participants who, due to cognitive impairment or other reasons, could not complete the questionnaire independently were excluded from the study. This short interview questionnaire is a validated tool for the assessment of depression (24). This is a questionnaire that inquired about the frequency of depressive symptoms in the previous 2 weeks (25). The nine-item instrument response categories “not at all,” “a few days,” “more than half of the days” and “almost every day” were scored (range 0–3). Each participant’s PHQ score is the total of all responses to the PHQ questions (range 0–27). A cut-off point of ≥10 indicates 88% sensitivity and specificity for diagnosing major depressive disorder (26). Therefore, we divided the participants into depression (PHQ-9 ≥ 10) and no depression (PHQ-9 < 10).

### 2.3 Exposure measurement

Participants measured isometric grip strength using a handgrip dynamometer. After the practice, participants were required to squeeze the dynamometer as hard as they could at random with their dominant or non-dominant hand and then repeat the test for the other hand. Every hand was examined three times, exchanging between trials and resting for 1 min between each measurement of the same hand. Participants who had severe upper limb disabilities or paralysis preventing them from gripping the dynamometer with both hands were excluded from the study. Body mass index (BMI) was computed using weight in kilograms divided by height in meters squared (kg/m^2^). Relative grip strength was calculated as absolute grip strength (maximum sum of readings for each hand) divided by BMI, and expressed in kg/BMI.

### 2.4 Assessment of other covariates

The selection of covariates was based on previous literature and clinical experience (27–29). Standardized questionnaires were designed to obtain information about age, gender, race, education level, poverty to income ratio (PIR), marital status, smoking and drinking status, moderate or vigorous activity, and sleep duration. Race was classified as Mexican American, other Hispanic, non-Hispanic white, non-Hispanic black, or other. Education level was categorized as did not graduate from high school, graduated from high school, and college education or above. Marital status was reported as married, widowed, divorced, separated, never married, and living with a partner. Smoking and drinking status were created from questions: “smoked at least 100 cigarettes in life (yes or no)” and “had at least 12 alcohol drinks a year (yes or no).” Moderate or vigorous activity was defined as moderate or vigorous activity usually performed for at least 10 min and resulting in a moderate or vigorous increase in respiration or heart rate.

The Glycohemoglobin (HbA1c), total cholesterol (TC), and white blood cell count (WBC) were measured according to standardized protocols. Physician-diagnosed medical co-morbidities, including cardiovascular diseases (angina, coronary heart diseases, heart attack, or congestive heart failure), stroke, thyroid problem, liver condition, cancer or malignancy, weak/failing kidneys, hypertension were identified by asking participants, “Has a doctor or other health professional ever told that you have diseases mentioned above.”

### 2.5 Statistical analysis

All continuous variables with normal distribution and skewness were expressed as mean (SD) or median (interquartile range [IQR]). Categorical variables were indicated as frequencies (%). The baseline characteristics of different relative grip strength groups were analyzed using the One-Way ANOVA (normal distribution), Kruskal-Wallis H (skewed distribution), and chi-square test (categorical variables). The effect of incident depression on relative grip strength was evaluated using logistic regression models (odds ratios [OR] and 95% confidence interval [CI]). In this study, we used unadjusted and multivariate-adjusted models. Model 1 was adjusted for sociodemographic characteristics, including age, gender, race, educational level, PIR, and marital status. Model 2 was adjusted for co-morbidities, including cardiovascular disease, stroke, thyroid problem, liver condition, cancer or malignancy, weak/failing kidneys, and hypertension. Model 3 was completely adjusted, including sociodemographic characteristics, co-morbidities, smoking and drinking status, moderate or vigorous activity, sleeping time, HbA1c, TC, and WBC.

In addition, a generalized additive model was applied to assess the nonlinear relationship between relative grip strength and incident depression in the logistic regression Model 3. Based on the smoothing curve, we further analyzed the association thresholds using a two-piece logistic regression model between relative grip strength and incident depression after adjusting Model 3. When the ratio between incident depression and relative grip strength was displayed obviously in a smoothed curve, the recursive method would calculate the inflection points automatically, and a maximum model likelihood would be applied here.

Moreover, potential modifications of the relationship between relative grip strength and incident depression were assessed, including age, gender, race, and hypertension. Subgroup analyses were evaluated by multivariate logistic regression models. Interactions between subgroups and relative grip strength were tested using the likelihood ratio test. To assess the robustness of the findings, participants with extreme relative grip strength outside the mean ± 3 SD (0–4.636 kg/BMI) were excluded, for sensitivity analyses.

All analyses were conducted using the statistical software packages R and Free Statistics software version 1.7. A two-sided *p* value <0.05 was regarded as having statistical significance.

## 3 Results

### 3.1 Study population

**Figure 1:**
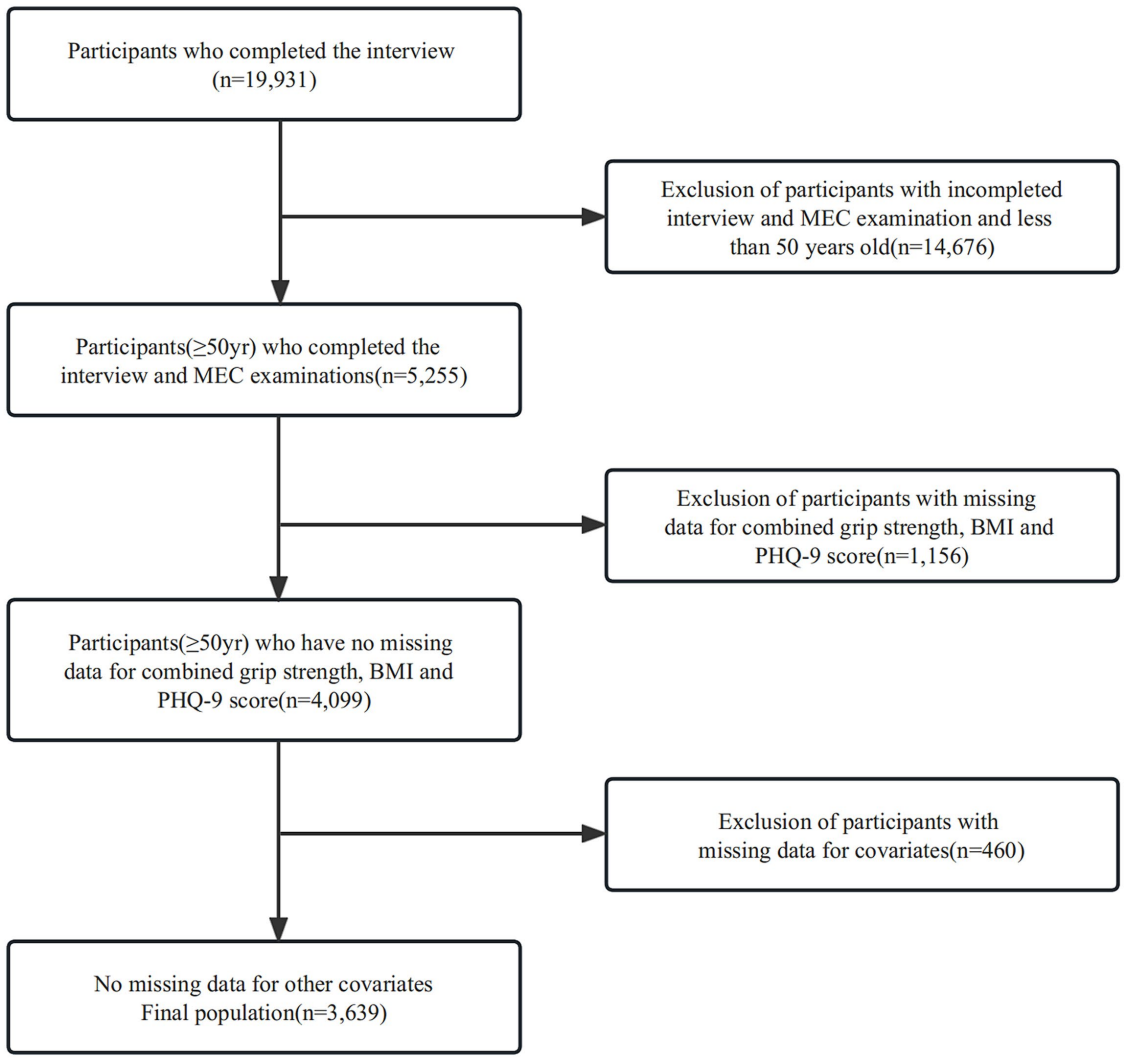
Participants inclusion flowchart.

This study included 19,931 prospective participants from NHANES (2011–2014), of which 5,255 middle-aged and older adults (≥50 years) completed interviews and were subjected to MEC screening. Participants with missing data for combined grip strength, BMI, and PHQ-9 scores (*n* = 1,156) were excluded. Our cross-sectional study included 3,639 participants after the exclusion of those with missing covariate data (*n* = 460) eventually. The study population selection flowchart is shown in Figure 1.

### 3.2 Baseline characteristics

The mean participant age was 64.3 ± 9.3 years, and 1,781 (48.9%) were men. The mean baseline Relative grip strength was 2.3 ± 0.8. There were 343 (9.4%) participants with depression. The detailed characteristics of the population by Relative grip strength quartiles are available in Table 1. Participants with higher relative grip strength were typically male and younger. They tended to have higher levels of education and income, engage more in smoking, alcohol consumption, and physical activity, and have shorter sleep duration. Additionally, participants with higher relative grip strength had lower rates of comorbidities and lower levels of HbA1c, TC, and WBC. The depression prevalence in this study was 9.4% (343 participants), which was, respectively, 15.9% (145 participants), 10.9% (99 participants), 5.4% (49 participants), 5.5% (50 participants) in relative grip strength quartile 1–4 (*p* < 0.001).

**Table 1:** Baseline characteristics of the study population.

Variables	Total (*n* = 3,639)	Q1 (*n* = 910)	Q2 (*n* = 909)	Q3 (*n* = 906)	Q4 (*n* = 914)	*p*-value
Age, (years)	64.3 ± 9.3	67.1 ± 9.7	64.5 ± 9.0	64.4 ± 9.2	61.0 ± 8.1	<0.001
Gender, *n* (%)						<0.001
Male	1,781 (48.9)	91 (10)	239 (26.3)	588 (64.9)	863 (94.4)	
Female	1,858 (51.1)	819 (90)	670 (73.7)	318 (35.1)	51 (5.6)	
Race, *n* (%)						<0.001
Mexican American	321 (8.8)	92 (10.1)	85 (9.4)	76 (8.4)	68 (7.4)	
Other Hispanic	341 (9.4)	108 (11.9)	97 (10.7)	77 (8.5)	59 (6.5)	
Non-Hispanic White	1,724 (47.4)	460 (50.5)	421 (46.3)	441 (48.7)	402 (44)	
Non-Hispanic Black	868 (23.9)	191 (21)	212 (23.3)	203 (22.4)	262 (28.7)	
Other Race	385 (10.6)	59 (6.5)	94 (10.3)	109 (12)	123 (13.5)	
Education level, *n* (%)						<0.001
Did not graduate from high school	841 (23.1)	253 (27.8)	204 (22.4)	190 (21)	194 (21.2)	
Graduated from high school	1,898 (52.2)	516 (56.7)	495 (54.5)	448 (49.4)	439 (48)	
College education or above	900 (24.7)	141 (15.5)	210 (23.1)	268 (29.6)	281 (30.7)	
PIR	2.3 (1.2, 4.4)	1.8 (1.0, 3.4)	2.2 (1.2, 4.2)	2.6 (1.3, 5.0)	3.0 (1.3, 5.0)	<0.001
Marital status, *n* (%)						<0.001
Married	2,017 (55.4)	401 (44.1)	470 (51.7)	544 (60)	602 (65.9)	
Widowed	507 (13.9)	224 (24.6)	148 (16.3)	88 (9.7)	47 (5.1)	
Divorced	551 (15.1)	160 (17.6)	147 (16.2)	141 (15.6)	103 (11.3)	
Separated	134 (3.7)	28 (3.1)	43 (4.7)	23 (2.5)	40 (4.4)	
Never married	318 (8.7)	77 (8.5)	79 (8.7)	82 (9.1)	80 (8.8)	
Living with partner	112 (3.1)	20 (2.2)	22 (2.4)	28 (3.1)	42 (4.6)	
Smoking status, *n* (%)						<0.001
Smoked at least 100 cigarettes	1,834 (50.4)	375 (41.2)	423 (46.5)	471 (52)	565 (61.8)	
Drinking status, *n* (%)						<0.001
≥12 alcohol drinks a year	2,599 (71.4)	504 (55.4)	621 (68.3)	678 (74.8)	796 (87.1)	
Moderate or vigorous activity, *n* (%)	1,238 (34.0)	216 (23.7)	302 (33.2)	322 (35.5)	398 (43.5)	<0.001
Sleeping time, (hours)	6.9 ± 1.4	7.0 ± 1.6	6.9 ± 1.5	6.9 ± 1.3	6.8 ± 1.3	0.017
**Co-morbidities**						
Cardiovascular diseases, *n* (%)	486 (13.4)	163 (17.9)	113 (12.4)	115 (12.7)	95 (10.4)	<0.001
Stroke, *n* (%)	215 (5.9)	85 (9.3)	52 (5.7)	38 (4.2)	40 (4.4)	<0.001
Thyroid problem, *n* (%)	549 (15.1)	236 (25.9)	169 (18.6)	94 (10.4)	50 (5.5)	<0.001
Liver condition, *n* (%)	192 (5.3)	64 (7)	47 (5.2)	35 (3.9)	46 (5)	0.025
Cancer or Malignancy, *n* (%)	578 (15.9)	157 (17.3)	140 (15.4)	144 (15.9)	137 (15)	0.576
Weak/Failing kidneys, *n* (%)	175 (4.8)	66 (7.3)	42 (4.6)	32 (3.5)	35 (3.8)	< 0.001
Hypertension	2,016 (55.4)	637 (70)	514 (56.5)	444 (49)	421 (46.1)	<0.001
HbA1c, (%)	6.0 ± 1.2	6.2 ± 1.2	6.1 ± 1.4	6.0 ± 1.0	5.8 ± 1.0	<0.001
TC, (mg/dL)	195.6 ± 43.6	196.9 ± 45.1	199.8 ± 44.4	193.3 ± 43.0	192.2 ± 41.5	<0.001
WBC, (1,000 cells/uL)	7.0 ± 2.4	7.3 ± 2.7	7.0 ± 2.6	6.8 ± 1.9	6.7 ± 2.2	<0.001
Depression, *n* (%)	343 (9.4)	145 (15.9)	99 (10.9)	49 (5.4)	50 (5.5)	<0.001
Relative grip strength, (kg/BMI)	2.3 ± 0.8	1.3 ± 0.2	1.9 ± 0.2	2.5 ± 0.2	3.4 ± 0.4	<0.001

Data were mean ± SD or median (IQR) for skewed variables or numbers (proportions) for categorical variables. PIR, ratio of family income to poverty; HbA1c, Glycohemoglobin; TC, total cholesterol; WBC, white blood cell count; Q1, Q2, Q3, and Q4 are quartiles of the relative grip strength.

### 3.3 Association between relative grip strength and depression

The univariate analysis demonstrated that age, gender, education level, PIR, smoking status, sleeping time, cardiovascular disease, stroke, liver condition, weak/failing kidneys, hypertension, HbA1c, TC, WBC, and relative grip strength were associated with depression (Supplementary Table S2).

**Figure 2:**
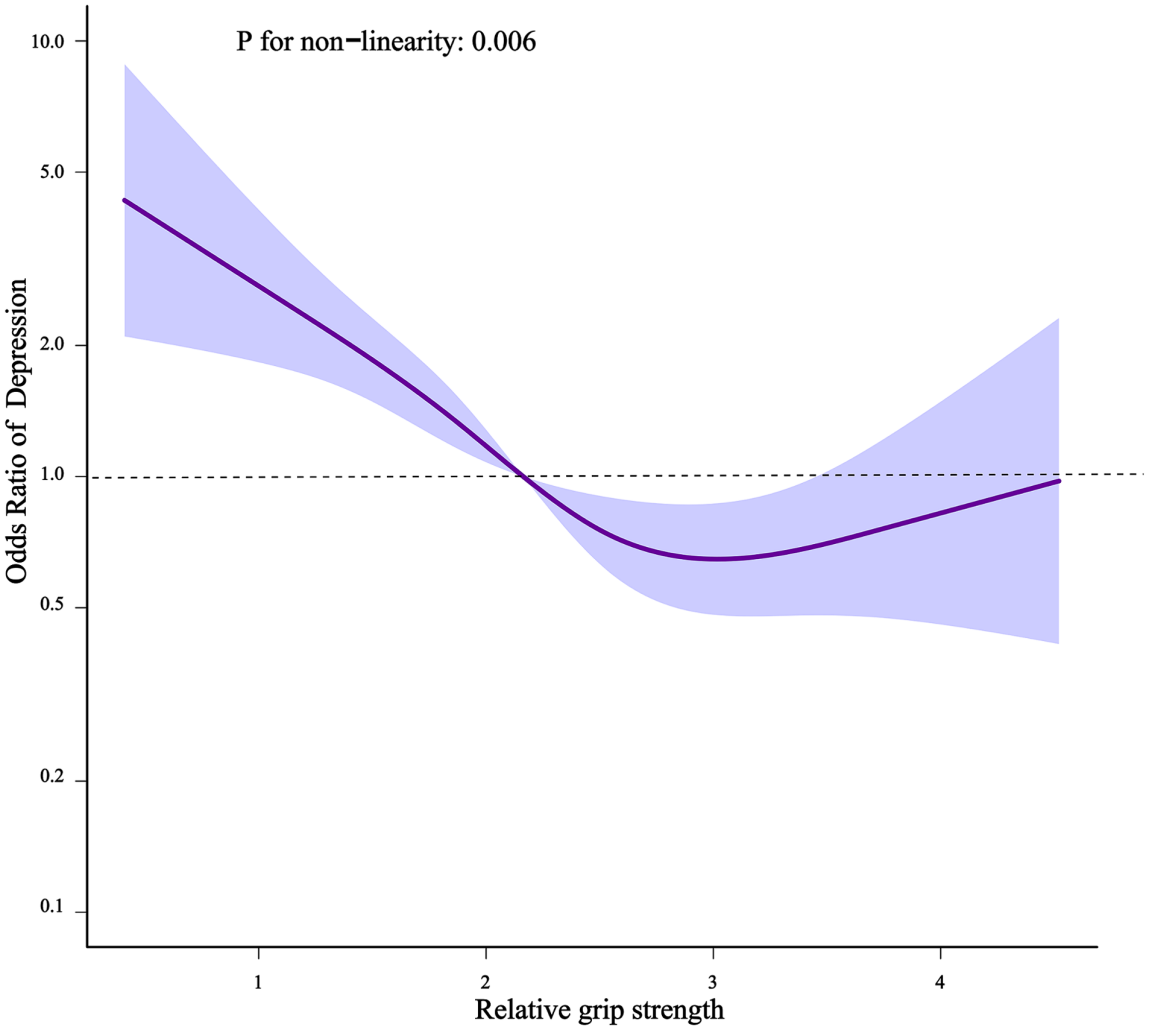
Non-linear relationship of relative grip strength and depression.

When relative grip strength was assessed as a continuous variable, the adjusted OR was 0.59 (95% CI: 0.47–0.73) for depression in the full variables adjusted model (model 3). When relative grip strength was analyzed using quartiles, a significant inverse relationship was found between relative grip strength and depression after adjusting the full variables. Compared with participants with lower relative grip strength in Q1 (≤1.64 kg/BMI), the adjusted OR values for relative handgrip strength and depression in Q2 (1.64–2.17 kg/BMI), Q3 (2.17–2.84 kg/BMI), and Q4 (≥2.84 kg/BMI) were 0.69 (95% CI: 0.51, 0.93, *p* = 0.016), 0.36 (95% CI: 0.24, 0.55, *p* < 0.001), 0.32 (95% CI: 0.20, 0.51, p < 0.001), respectively. All of the models were statistically significant (Table 2, *p* for trend < 0.05). In addition, the relationship between relative grip strength and depression exhibited an L-shaped curve (nonlinear, *p* = 0.006) after adjusting for all covariates (Figure 2). In the threshold analysis, the OR of developing depression was 0.41 (95% CI: 0.30, 0.55, *p* < 0.001) in participants with relative grip strength < 2.98 kg/BMI (Table 3). This means that the risk of depression is reduced by 59% with every 1 kg/BMI increase. The relationship between relative grip strength and depression was not observed when the relative grip strength was ≥2.98 kg/BMI (Table 3). This suggests that incident depression no longer decreases with increasing relative grip strength.

**Table 2:** Multivariable-adjust ORs and 95%CI of the relative grip strength quartiles associated with depression.

Variable	Unadjusted		Model 1		Model 2		Model 3	
	OR (95%CI)	*p*-value	OR (95%CI)	*p*-value	OR (95%CI)	*p*-value	OR (95%CI)	*p*-value
Relative grip strength	0.54 (0.46~0.63)	<0.001	0.54 (0.44~0.67)	<0.001	0.61 (0.49~0.75)	<0.001	0.59 (0.47~0.73)	<0.001
1st Quartile(≤1.64)	1(Ref)		1(Ref)		1(Ref)		1(Ref)	
2st Quartile(1.64–2.17)	0.64 (0.49~0.85)	0.002	0.65 (0.48~0.87)	0.003	0.72 (0.53~0.97)	0.031	0.69 (0.51~0.93)	0.016
3st Quartile(2.17–2.84)	0.3 (0.22~0.42)	<0.001	0.31 (0.21~0.47)	<0.001	0.37 (0.25~0.56)	<0.001	0.36 (0.24~0.55)	<0.001
4st Quartile(≥2.84)	0.31 (0.22~0.43)	<0.001	0.28 (0.18~0.45)	<0.001	0.34 (0.21~0.54)	<0.001	0.32 (0.20~0.51)	<0.001
*P* for trend		<0.001		<0.001		<0.001		<0.001

Model 1 adjust for Age, Gender, Race, Education level, PIR, Marital status. Model 2 adjust for Model 1 + Cardiovascular diseases, Stroke, Thyroid problem, Liver condition, Cancer or Malignancy, Weak/Failing kidneys, Hypertension. Model 3 adjust for Model 1 + Model 2 + Smoking status, Drinking status, Moderate or vigorous activity, Sleeping time, HbA1c, TC, WBC. Ref, reference; PIR, ratio of family income to poverty; HbA1c, Glycohemoglobin; TC, total cholesterol; WBC, white blood cell count.

**Table 3:** Threshold effect analysis of the relationship of relative grip strength with depression.

Relative grip strength (kg/BMI)	Adjusted model	
	OR (95%CI)	*p*-value
<2.98	0.41 (0.30~0.55)	<0.001
≥2.98	1.25 (0.59~2.65)	0.562
Likelihood ratio test		0.011

OR, odds ratio; CI, confidence interval. Adjusted for Model 3.

In Figure 2, the purple solid line represents the estimated risk of incident depression, and the light blue shadow represents a point-wise 95% confidence interval adjusted for age, gender, race, educational level, PIR, marital status, smoking and drinking status, moderate or vigorous activity, sleeping time, cardiovascular diseases, stroke, thyroid problem, liver condition, cancer or malignancy, weak/failing kidneys, hypertension, HbA1c, TC, WBC.

### 3.4 Stratified analyses based on additional variables

**Figure 3:**
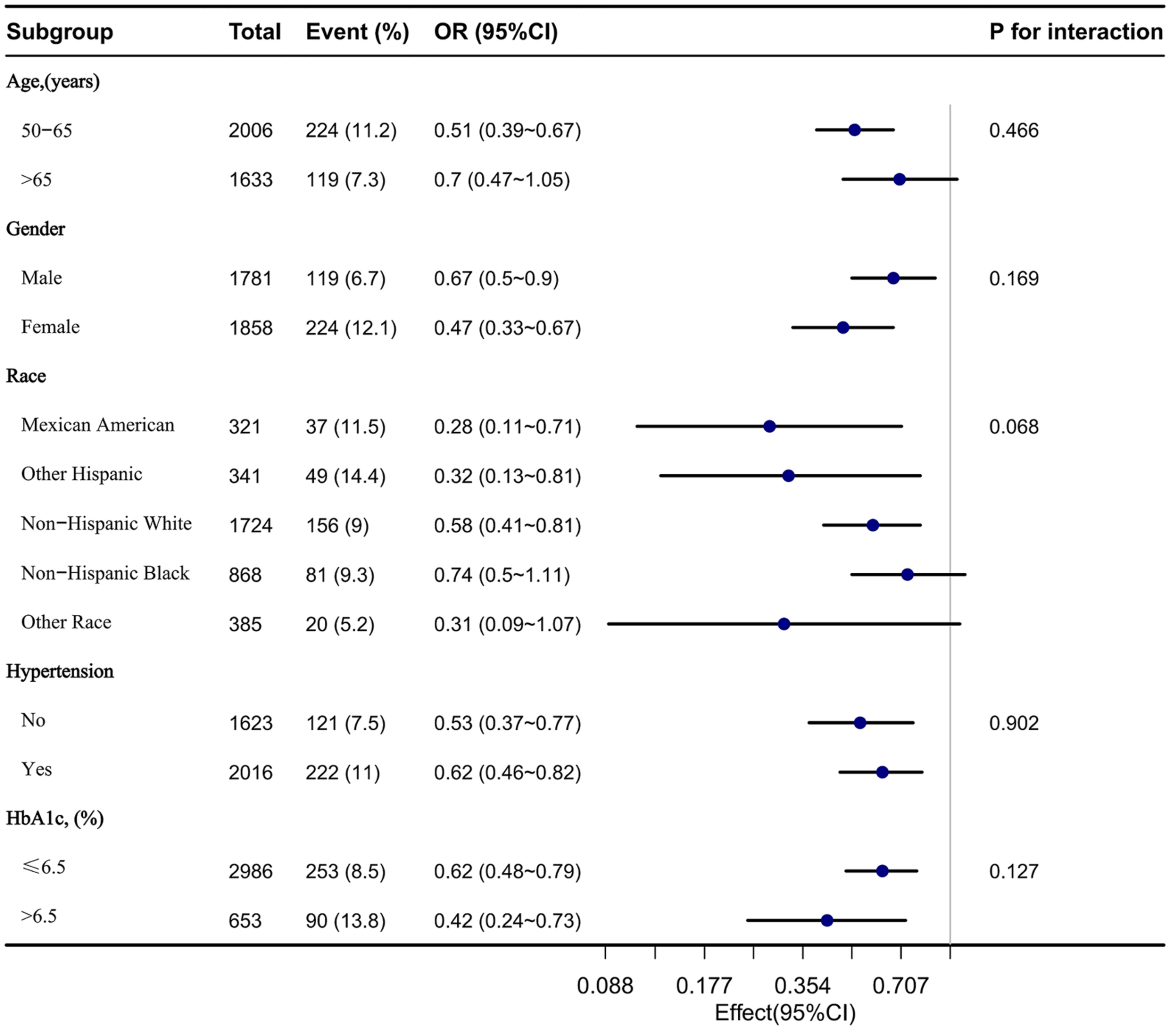
The relationship between relative grip strength and depression according to basic features.

Stratified analyses were performed in several subgroups to assess the potential effect modification of the relationship between relative grip strength and depression. Significant interactions were not observed in any of the subgroups after stratifying by age, gender, race, hypertension, and HbA1c (Figure 3).

### 3.5 Sensitivity analysis

3,627 participants were left after excluding the participants with extreme relative grip strength, and the association between relative grip strength and incident depression remained stable. As compared to participants with lower relative grip strength in Q1 (≤1.64 kg/BMI), the adjusted OR values for relative grip strength and depression in Q2 (1.64–2.17 kg/BMI), Q3 (2.17–2.84 kg/BMI), and Q4 (≥2.84 kg/BMI) were 0.68 (95% CI: 0.50, 0.93, *p* = 0.015), 0.37 (95% CI: 0.25, 0.56, *p* < 0.001), and 0.32 (95% CI: 0.20, 0.51, *p* < 0.001), respectively (Supplementary Table S2).

## 4 Discussion

As far as we know, there are no other studies investigating the relationship between relative grip strength and depression among U.S. middle-aged and older adults. In this large population-based cross-sectional study of United States middle-aged and older adults (≥50 years) using 2011–2014 NHANES data, it was found that there was a negative association between relative grip strength and incident depression when adjusted for potential confounding factors. The relationship between relative grip strength and incident depression remained robust after stratified and sensitivity analyses were performed. We further revealed an L-shaped relationship between relative grip strength and incident depression, with an inflection point of almost 2.98 kg/BMI. This indicated that within a certain range, lower relative grip strength was significantly associated with a higher risk of depression.

Poor muscle strength has often been thought to increase the risk of depression in older adults (30, 31) and grip strength can be an appropriate representation of overall muscle strength (4). However, previous studies on the relationship between absolute grip strength and depression have yielded conflicting results (11, 12, 32), possibly due to the lack of consideration of body size factors. Individuals with obesity have significantly lower relative strength compared to absolute strength, thus it is essential to fully consider the impact of body size when assessing muscle strength (33, 34). Previous studies have indicated that relative grip strength demonstrates higher sensitivity to low mobility and poor health status in older adults compared to muscle strength or BMI (20, 35, 36). Our study further provided evidence of the impact of relative strength on mental health.

Several potential mechanisms, both psychological and physiological, could elucidate the connection between lower relative grip strength and an increased risk of depression. In older adults, reduced relative grip strength often correlates with a decline in physical function, impacting their ability to accomplish daily tasks effectively (37). Physiologically, oxidative stress and inflammation are hypothesized mechanisms linking muscle strength to mental health (38). Research has highlighted inflammation as a contributor to muscle mass decline in older individuals (39), with inflammation-induced cytokines and reactive oxygen species implicated in sarcopenia and muscle aging (40, 41). These factors potentially heighten the susceptibility to depression (42). Furthermore, myokines released during muscle contraction may play a protective role against depression (43). However, more prospective studies are required to confirm the appropriate range of relative grip strength to prevent chronic diseases in older adults.

We also discovered an L-shaped relationship between relative grip strength and depression. The appearance of an L-shaped relationship indicated that beyond a certain level of relative grip strength, further increases in relative grip strength ceased to significantly affect the risk of depression. One possible explanation for this phenomenon was that when individuals had already achieved relatively good physiological and psychological health, additional increases in muscle strength gradually diminished in their impact on mental health. Additionally, other confounding factors such as education level and marital status may played a greater role in depression at higher levels of relative grip strength. Nevertheless, relative grip strength, as a convenient measure addressing the confounding effect of body size on strength, warranted more attention.

The strengths of our study included adjusting for multiple co-morbidities and biochemical indicators that were closely associated with depression (11, 24, 44) and the relatively large sample size. This study also has some limitations. Firstly, grip strength data were only collected in NHANES from 2011 to 2014. Therefore, we were unable to use data from other time periods to further validate the results, which may have introduced some bias into the findings. Secondly, due to the inherent limitations of cross-sectional studies, the causal relationship between relative grip strength and depression cannot be determined, and current relationships may be bidirectional (45). Thirdly, our study relied on previously collected data, thus potentially introducing bias by not comprehensively incorporating confounding factors such as diet and medication use. Fourthly, information on co-morbidities, such as cardiovascular diseases, stroke, thyroid problem, liver condition, cancer or malignancy, weak/failing kidneys, and hypertension, was self-reported by participants, which potentially can lead to error. Finally, this study lacked an assessment of cognitive function, which could confound the relationship between grip strength and depression (46). Although studies had shown that grip strength was positively correlated with cognitive function in patients with affective disorders such as depression, this relationship persisted in healthy populations as well (47, 48). Future research should include cognitive assessments to conduct stratified analyses.

## 5 Conclusion

This study identified an L-shaped association between relative grip strength and the prevalence of incident depression among U.S. middle-aged and older adults (≥50 years), with an inflection point at approximately 2.98 kg/BMI. These findings suggest that lower relative grip strength has higher odds of incident depression up to a threshold, beyond which the association weakens. This relationship highlights the potential of using relative grip strength as a screening tool for depression risk in older adults. Future longitudinal research should explore the causal mechanisms underlying this association and investigate interventions to improve grip strength as a preventive measure against depression.

## Data availability statement

Publicly available datasets were analyzed in this study. All data can be found in the National Health and Nutrition Examination Survey Homepage: http://www.cdc.gov/nchs/nhanes/index.htm.

## Ethics statement

The studies involving humans were approved by the Institutional Review Board of the National Center for Health Statistics authorized the NHANES procedure. The studies were conducted in accordance with the local legislation and institutional requirements. The human samples used in this study were acquired from gifted from another research group. Written informed consent for participation was not required from the participants or the participants’ legal guardians/next of kin in accordance with the national legislation and institutional requirements.

## Author contributions

AS: Conceptualization, Data curation, Formal analysis, Methodology, Writing – original draft, Writing – review & editing. ZL: Conceptualization, Funding acquisition, Writing – review & editing.
