# Supplementary material for: Association between relative grip strength and depression among U.S. middle-aged and older adults: results from the NHANES database
Source: Front Public Health. 2024 Jul 29;12:1416804. doi: 10.3389/fpubh.2024.1416804 (PMC11317278; doi:10.3389/fpubh.2024.1416804)
Supplement: Supplementary file 1 [file Table_1.DOC]

**Table S1.**Results of univariate analysis of Depression.

| **Variable** | **OR(95%CI)** | **P-value** |
| --- | --- | --- |
| Age, (years) | 0.97 (0.96~0.99) | <0.001 |
| Gender, n (%) |  |  |
| Male | Ref |  |
| Female | 1.91 (1.52~2.42) | <0.001 |
| Race, n (%) |  |  |
| Mexican American | Ref |  |
| Other Hispanic | 1.29 (0.82~2.03) | 0.278 |
| Non-Hispanic White | 0.76 (0.52~1.12) | 0.164 |
| Non-Hispanic Black | 0.79 (0.52~1.19) | 0.262 |
| Other Race | 0.42 (0.24~0.74) | 0.003 |
| Education level, n (%) |  |  |
| Did not graduate from high school | Ref |  |
| Graduated from high school | 0.58 (0.45~0.74) | <0.001 |
| College education or above | 0.27 (0.19~0.39) | <0.001 |
| PIR | 0.65 (0.6~0.71) | <0.001 |
| Marital status, n (%) |  |  |
| Married | Ref |  |
| Widowed | 1.66 (1.19~2.3) | 0.003 |
| Divorced | 2.25 (1.67~3.02) | <0.001 |
| Separated | 3.12 (1.96~4.99) | <0.001 |
| Never married | 1.63 (1.1~2.42) | 0.015 |
| Living with partner | 1.79 (0.98~3.27) | 0.059 |
| Smoking status, n (%) |  |  |
| Smoked at least 100 cigarettes | 1.65 (1.31~2.07) | <0.001 |
| Drinking status, n (%) |  |  |
| ≥ 12 alcohol drinks a year | 1.08 (0.84~1.39) | 0.528 |
| Moderate or vigorous activity, n (%) | 0.79 (0.62~1.01) | 0.061 |
| Sleeping time, (hours) | 0.76 (0.7~0.82) | <0.001 |
| Co-Morbidities |  |  |
| Cardiovascular diseases, n (%) | 2.08 (1.59~2.74) | <0.001 |
| Stroke, n (%) | 2.27 (1.58~3.28) | <0.001 |
| Thyroid problem, n (%) | 1.3 (0.97~1.74) | 0.075 |
| Liver condition, n (%) | 2.1 (1.42~3.11) | <0.001 |
| Cancer or Malignancy, n (%) | 0.89 (0.65~1.22) | 0.487 |
| Weak/Failing kidneys, n (%) | 2.66 (1.81~3.91) | <0.001 |
| Hypertension | 1.54 (1.22~1.94) | <0.001 |
| HbA1c, (%) | 1.19 (1.1~1.28) | <0.001 |
| TC, (mg/dL) | 1 (1~1.01) | 0.028 |
| WBC, (1000 cells/uL) | 1.05 (1.01~1.09) | 0.007 |
| Relative grip strength, (kg/BMI) | 0.54 (0.46~0.63) | <0.001 |

Ref, reference; PIR, ratio of family income to poverty; HbA1c, Glycohemoglobin; TC, total cholesterol;

WBC, white blood cell count.
