# Supplementary material for: Association between relative grip strength and depression among U.S. middle-aged and older adults: results from the NHANES database
Source: Front Public Health. 2024 Jul 29;12:1416804. doi: 10.3389/fpubh.2024.1416804 (PMC11317278; doi:10.3389/fpubh.2024.1416804)
Supplement: Supplementary file 2 [file Table_2.DOC]

**Table S2.** Multivariable-adjust ORs and 95%CI of the Relative grip strength quartiles associated with Depression. Extreme relative grip strength was not included.

| **Variable** | **Unadjusted** | |  | **Model 1** | |  | **Model 2** | |  | **Model 3** | |
| --- | --- | --- | --- | --- | --- | --- | --- | --- | --- | --- | --- |
|  | **OR(95%CI)** | **P-value** |  | **OR(95%CI)** | **P-value** |  | **OR(95%CI)** | **P-value** |  | **OR(95%CI)** | **P-value** |
| Relative grip strength | 0.54 (0.46~0.63) | <0.001 |  | 0.54 (0.44~0.67) | <0.001 |  | 0.59(0.48~0.74) | <0.001 |  | 0.59 (0.47~0.74) | <0.001 |
| 1st Quartile(≤1.64) | 1(Ref) |  |  | 1(Ref) |  |  | 1(Ref) |  |  | 1(Ref) |  |
| 2st Quartile(1.64-2.17) | 0.64 (0.48~0.84) | 0.001 |  | 0.64 (0.48~0.86) | 0.003 |  | 0.69 (0.51~0.94) | 0.018 |  | 0.68 (0.50~0.93) | 0.015 |
| 3st Quartile(2.17-2.84) | 0.31 (0.22~0.43) | <0.001 |  | 0.32 (0.22~0.48) | <0.001 |  | 0.37 (0.25~0.56) | <0.001 |  | 0.37 (0.25~0.56) | <0.001 |
| 4st Quartile(≥2.84) | 0.30 (0.22~0.43) | <0.001 |  | 0.28 (0.18~0.45) | <0.001 |  | 0.32 (0.20~0.51) | <0.001 |  | 0.32 (0.20~0.51) | <0.001 |
| P for trend |  | <0.001 |  |  | <0.001 |  |  | <0.001 |  |  | <0.001 |

Model 1 adjust for Age, Gender, Race, Education level, PIR, Marital status.

Model 2 adjust for Model 1+Cardiovascular diseases, Stroke, Thyroid problem, Liver condition, Cancer or Malignancy, Weak/Failing kidneys, Hypertension.

Model 3 adjust for Model 1+Model 2+Smoking status, Drinking status, Moderate or vigorous activity, Sleeping time, HbA1c, TC, WBC.

Ref, reference; PIR, ratio of family income to poverty; HbA1c, Glycohemoglobin; TC, total cholesterol; WBC, white blood cell count.
